# Correlations between serum levels of microRNA-148a-3p and microRNA-485-5p and the progression and recurrence of prostate cancer

**DOI:** 10.1186/s12894-022-01143-z

**Published:** 2022-11-25

**Authors:** Wenyan He, Furong Zhang, Feng Jiang, Huan Liu, Gang Wang

**Affiliations:** 1 Department of Urology, Yan’an People’s Hospital, Yan’an, China; 2 Department of Neurology, Affiliated Hospital of Yan’an University, Yan’an, China; 3 Department of Medicine, Aab Cardiovascular Research Institute, University of Rochester School of Medicine and Dentistry, Rochester, NY USA

**Keywords:** Prostate cancer, miR-148a-3p, miR-485-5p, Gleason score, TNM staging, Recurrence

## Abstract

**Background:**

Unpredicted postoperative recurrence of prostate cancer, one of the most common malignancies among males worldwide, has become a prominent issue affecting patients after treatment. Here, we investigated the correlation between the serum miR-148a-3p and miR-485-5p expression levels and cancer recurrence in PCa patients, aiming to identify new biomarkers for diagnosis and predicting postoperative recurrence of prostate cancer.

**Methods:**

A total of 198 male PCa cases treated with surgery, postoperative radiotherapy, and chemotherapy were involved in the presented study. Serum levels of miR-148a-3p and miR-485-5p were measured before the initial operation for the involved cases, which were then followed up for two years to monitor the recurrence of cancer and to split the cases into recurrence and non-recurrence groups. Comparison of the relative expressions of serum miR-148a-3p and miR-485-5p were made and related to other clinic pathological features.

**Results:**

Pre-surgery serum levels of miR-148a-3p in patients with TNM stage cT1-2a prostate cancer (Gleason score < 7) were significantly lower (*P* < 0.05) than levels in patients with TNM Classification of Malignant Tumors (TNM) stage cT2b and higher prostate cancer (Gleason score ≥ 7). pre-surgery serum levels of miR-485-5p in patients with TNM stage cT1-2a prostate cancer (Gleason score < 7) were significantly higher (*P* < 0.05) than in patients with TNM stage cT2b and higher cancer (Gleason score ≥ 7). Serum miR-148a-3p level in recurrence group is higher than the non-recurrence group (*P* < 0.05) while serum miR-485-5p level in recurrence group is lower than non-recurrence group (*P* < 0.05). ROC curve analysis showed the AUCs of using miR-148a-3p, miR-485-5p, and combined detection for predicting recurrence of prostate cancer were 0.825 (95% CI 0.765–0.875, *P* < 0.0001), 0.790 (95% CI 0.726–0.844, *P* < 0.0001), and 0.913 (95% CI 0.865–0.948, *P* < 0.0001).

**Conclusion:**

Pre-surgery serum miR-148a-3p level positively correlates while miR-485-5p level negatively correlates with prostate cancer’s progressing and postoperative recurrence. Both molecules show potential to be used for predicting postoperative recurrence individually or combined.

## Background

As the fifth most frequent cause of male death worldwide, prostate cancer (PCa) counts for more than 30% of all newly diagnosed cancers with an approximately 5-year survival rate of 9% [1, 2]. Advanced prostate cancer exhibits signs and symptoms, including typically slow urination, difficulty emptying the bladder, blood in the urine, and back pain [1, 3]. Screening and diagnosis of PCa are important for detecting cases, predicting disease outcomes, guiding clinical management decisions, and avoiding overtreatment [4–6]. Thus, rapid sensitive diagnostic methods are in demand. Including measurements of serum prostate-specific antigens (PSA/KLK3) in the diagnosis of prostate cancer has led to a significant increase in the detection of early-stage PCa (Gleason < 6) [6–10]. However, the specificity and sensitivity of serum PSA are still not high enough for detecting early-stage prostate cancer or precisely evaluating the progression or severity of cancer since elevated PSA levels are also detected in benign prostatic hyperplasia, inflammation, and other urinary tract diseases [11]. Thus, sensitive and specific biomarkers for prostate cancer diagnosis are in high demand.

Apart from the difficulties in the early diagnosis of prostate cancer, postoperative recurrence is another major issue threatening the patients’ health and recovery. Postoperative recurrence is when the surviving prostate cancer cells become evident again after initial treatment, such as surgery or radiation therapy documenting the removal of cancer cells [12]. It occurs in around 15% of patients and always requires a second cancer treatment at least 6 months after surgery [13]. Clinically, the prediction of postoperative recurrence is based on serum prostate-specific antigens. A second increase following the initial drop-off after surgery or radiation therapy usually indicates the recurrence of prostate cancer cells. However, due to the low sensitivity and specificity of the currently used prostate-specific antigens, predicting cancer recurrence faces the same difficulty as early diagnosis of prostate cancer. Therefore, identifying new biomarkers is in need.

Recent studies have reported the potential of microRNAs (miRNAs) as diagnostic biomarkers and therapeutic targets of PCa. MicroRNAs are small single-strand non-coding RNA molecules regulating gene expression through complementary base pairing with target mRNAs, affecting the post-transcription processing of ~ 60% of the human genome through base-pairing with target mRNAs [6, 14]. The involvement of miRNAs in the development and progression of numerous tumors has been widely reported, highlighting the potential for cancer diagnosis and treatments [15]. The Association of miRNAs with prostate cancers was initially reported by a large-scale miRNA analysis using a large collection of samples, including prostate cancers [6]. At least 12 miRNAs were identified as overexpressed in prostate cancer, and further studies confirmed the role of miRNAs as new biomarkers for prostate cancer [16]. After that, numerous reports have suggested the essential roles of microRNAs (miRNAs) in PCa formation and progression [17]. The expression of those miRNAs constantly changes significantly during prostate tumors, indicating their potential as clinical biomarkers [18, 19]. miR-148a-3p and miR-485-5p are miRNAs showing particular diagnostic potential related to prostate cancer. miR-148a-3p, located on the 7p15.2 region of human chromosomes, has a well-established role in developing tumors [20]. Published studies suggested miR-148a-3p significantly decreased in various cancer cells, such as bladder cancer cells [21], suggesting its potential as a cancer biomarker. miR-485-5p, on the other hand, has been identified as a tumor suppressor with a significant inhibitory effect on the proliferation and differentiation of gastric cancer [22, 23]. miR-485-5p inhibits the expression of hypoxia-inducible factor 1 and impedes hepatocellular carcinoma cell differentiation, inhibiting the growth of liver cancer cells [24]. However, whether miR-148a-3p and miR-485-5p participate in the progression of prostate cancers is still unknown. Their potential as a biomarker for diagnosis and predicting prostate cancer recurrence has not been examined.

In the presented study, we examined the changes in serum miR-148a-3p and miR-485-5p levels in patients with prostate cancer at different stages and recurrence. Evaluated the correlations between serum miR-148a-3p and miR-485-5p levels and cancer progression and recurrence, aiming to identify new diagnostic biomarkers for prostate cancer.

## Materials and methods

### Study population

Retrospectively, this study was conducted on patients (aged 64.05 ± 10.11) with prostate cancer admitted to the Department of Urology, Yan’an People’s Hospital. Clinical data of patients were collected following approved protocols of the Committee of Yan’an People’s Hospital with written informed agreements obtained from patients. All patients showed the best adherence to the protocol. Informed consent was obtained from all patients. A total of 198 cases were enrolled and all cases satisfied the following criteria.

Inclusion criteria: (1) diagnosed with prostate cancer by surgical histopathological examination; (2) no history of prostate surgery; (3) with a traceable clinical history.

Exclusion criteria: (1) with severe prostatic hyperplasia; (2) with another malignant tumor; (3) with severe urinary system disease; (4) with coagulation dysfunction.

### Treatment and follow-up

All involved cases underwent radical prostatectomy. Radiotherapy (adjuvant radiotherapy for patients with low or intermediate risk) were routinely given according to China guideline for the screening and early detection of prostate cancer (2022, Beijing) [25]. The cases were follow-up postoperatively for two years. Re-examining serum PSA, ultrasonography and MRI were given once every 2 months. Recurrence or metastasis was determined based on the following criteria: (1) Continuous serum PSA ≥ 0.2 ng/mL; (2) MRI Diffusion-Weighted Imaging Sequences find other organs (mainly bone) have an obvious abnormal high signal shadow. Forty-five cases were determined as recurrence, while 153 cases as non-recurrence.

### Clinical data collection

The clinical history data collected includes age, body mass index, hypertension, diabetes, smoking history, and family history of prostate cancer. According to EAU guidelines 2022, preoperative Gleason scores were used to evaluate the risk of Pca (low-risk PCa: Gleason < 7, high-risk PCa: Gleason ≥ 7) with the following criteria: serum PSA level: low-risk PCa (≤ 10ng/mL), intermediate/high-risk PCa (> 10ng/mL), metastasis (TNM) classification for the staging of PCa: low-risk PCa (cT1-2a), intermediate/high-risk PCa (cT2b and higher), index lesion diameter: low-risk PCa (no index ), intermediate/high-risk PCa (≤ 7 mm ).

### Measurement of serum prostate-specific antigen levels

Serum levels of PSA (Human KLK3/PSA (Sandwich ELISA) ELISA Kit; Catalog#: LS-F22855-1; Detection Range: 0.938-60 ng/ml; Intra-assay: CV% <10%; Inter-assay CV% <10%) were measured according to the manufacturer’s instructions.

### Measurement of serum miR-148a-3p, mir-485-5p levels

#### Reagents and equipment

RNA extraction reagent: TRI Reagent® BD (TB 126), Catalog # TB126, Interassay CV: <10%, Intraassay CV: <10%, MRCGENE. Real-time catastrophe quantitative PCR kit: One-Step TB Green PrimeScript RT-PCR Kit II (Perfect Real Time), Catalog # RR086B, Interassay CV: <10%, Intraassay CV: <10%, takarabio. Reverse transcription kit: Efficient preparation of cDNA: PrimeScript Reverse Transcriptase, Catalog # 2680B, Interassay CV: <10%, Intraassay CV: <10%, takarabio. Wizard2 2-Detector Gamma Counter, 550 samples, Catalog # C 2470-0020, PerkinElmer, Germany. PCR instrument: ABI Geneamp 9700 PCR - Thermal Cycler, Catalog # ABI-97, ABI, USA. NanoDrop™ 2000/2000c Spectrophotometers, Catalog # ND2000CLAPTOP, Thermo Fisher Scientific, USA.

#### Specimen collection

Before the operation, 5 ml of fasting venous blood was collected and placed in an anticoagulation tube, centrifuged at 2500 rpm for 10 min to collect supernatants. All samples are stored at − 80 °C.

#### Determination of serum miR-148a-3p, miR-485-5p levels

Total RNA was extracted from 200 µl aliquoted plasma using an RNA extraction reagent. ND2000C UV spectrophotometer was used to determine the concentration and purity of the RNA. RNA samples with A260/A280 between 1.8–2.1 were used for cDNA synthesis by reverse transcription kit. miRNAs relatively levels were measured through RT-qPCR performed using the Real-time catastrophe quantitative PCR kit according to the following setup: PCR reaction system: cDNA template 3.0 µL, Premix ExTqTM 10 µL, upstream primer 1 µL, downstream primer 1 µL, and ddH2O 5.0 µL. Reaction conditions: 94 °C 5 min, 94 °C for 30 s, 58 °C for 30 s, 72 °C for 10 min, 40 cycles. Technical triplicates were performed. GAPDH and U6 were measured as internal controls to calculate the relative expression levels of miR-148a-3p and miR-485-5p.

For miRNAs measurements, the primer design and synthesis were completed by Guangzhou Ruibo Biotechnology Co., Ltd. miR-148a-3p: Forward: AGC TCT GCT ACT GAG ATG CG, Reverse: GAC TGC CAG CTA TCA TCG; miR-485-5p: Forward CTG GAA CGG TGA AGG TGA CA; Reverse: AAG GGA CTT CCT GTA ACA ACG CA; U6 Forward: GCT TCG GCA GCA CAT ATA CTA AAA T, Reverse: CGC TTC ACG AAT TTG CGT GTC AT. GAPDH Forward: AAG GTG AAG GTC GGA GTC A ; Reverse: GGA AGA TGG TGA TGG GAT TT.

### Statistical analysis

SPSS 22.0 software was used for statistical analysis. The data were expressed as the mean ± standard deviation ($\bar{\chi }$ ± *s*), and a comparison between the two groups was performed. Independent samples t-test was used. Count data were compared using the χ^2^ test. Predict receiver operating characteristic (ROC) model curve was used to evaluate the sensitivity and specificity of miR-148a-3p and miR-485-5p as biomarkers. *P* < 0.05 was considered to show a statistically significant difference.

## Results

### Characteristics of the study population

The baseline clinical characteristics of the study population are summarized in Table 1. Briefly, there is no statistically significant difference (*P* > 0.05) in age, body mass index, hypertension, diabetes, smoking history, and family history of PCa between recurrence and non-recurrence groups. Notably, the average age of the involved cases is 64.05 ± 10.11 years, the average age of patients with non-recurrence is 63.8 ± 8.4 years, and the average age of patients with recurrence is 64.0 ± 8.1 years.

**Table 1:** Clinical characteristics and perioperative data

Parameters	Non-recurrence group (n = 153)	Recurrence group (n = 45)	t/ χ^2^	*P*
Age (years)	63.8 ± 8.4	64.0 ± 8.1	0.638	0.524
BMI (kg/m^2^)	22.3 ± 2.4	22.5 ± 2.6	1.554	0.152
Hypertension (%)	49 (32.02)	14 (31.11)	0.095	0.763
Diabetes (%)	17 (11.11)	4 (8.89)	0.157	0.741
Smoking history (%)	84 (54.90)	19 (42.22)	3.548	0.052
The family history of PCa (%)	9 (5.88)	2 (4.44)	1.146	0.288

Data are shown as means ± SD. BMI, Body mass index; *χ*^*2*^*/t*, results of chi-square test; *P*-value, the result of student’s T-test. *P* < 0.05 is considered to be significant

### Serum miR-148a-3p increases while miR-485-5p decreases in the late stages of prostate cancer compared with earlier stages

We first examined the correlation between pre-surgery serum levels of miR-148a-3p and miR-485-5p and other clinical feathers in the involved cases before any operation (Table 2). We compared the level of miR-148a-3p and miR-485-5p in patients with different stages based on either TNM or Gleason scores. According to TNM staging, the involved cases were separated into two groups: 150 cases in the cT1-2a stage, 48 cases in cT2b, and higher stages. Pre-surgery serum levels of miR-148a-3p in patients at the cT1-2a stage (4.31 ± 2.01) were significantly lower than in patients at cT2b or higher stages (8.52 ± 2.28), while pre-surgery serum levels of miR-485-5p in patients at cT1-2a stage (4.02 ± 1.25) were significantly higher than patients at cT2b or higher stage (0.42 ± 0.28). According to the Gleason score, the involved cases were divided into two groups: 143 Gleason < 7 cases and 55 Gleason ≥ 7 cases. Pre-surgery serum levels of miR-148a-3p in Gleason < 7 cases (3.98 ± 1.25) were dramatically lower than Gleason ≥ 7 cases (7.05 ± 3.47 while pre-surgery serum levels of miR-485-5p in Gleason < 7 cases (1.40 ± 0.58) were significantly higher than Gleason ≥ 7 cases (0.52 ± 0.23). Both serum levels of miR-148a-3p and miR-485-5p show no significantly different (*P* > 0.05) related to the age (≥ 60 years compared with < 60 years old), index lesion diameter (no index compared with index lesion(s) ≤ 7 mm), or serum PSA level (> 10ng/mL compared with ≤ 10ng/mL).

**Table 2:** The levels of miR-148a-3p and miR-485-5p in preoperative serum of PCa patients

Parameters	Number (n)	miR-148a-3p	t	*P*	miR-485-5p	t	*P*
*Age*			0.525	0.584		0.952	0.350
≥ 60 years	127	4.85 ± 1.66			1.12 ± 0.54		
< 60 years	71	4.73 ± 1.58			1.18 ± 0.41		
*Index lesion diameter*			0.341	0.772		1.525	0.080
No index	103	4.85 ± 1.63			1.10 ± 0.44		
≤ 7 mm	95	4.77 ± 1.82			1.18 ± 0.51		
*PSA level*			0.711	0.500		1.915	0.083
> 10ng/mL	165	4.85 ± 2.12			1.11 ± 0.51		
≤ 10ng/mL	33	4.66 ± 1.98			1.32 ± 0.62		
*Gleason score*			7.520	< 0.001		17.58	< 0.001
< 7 points	143	3.98 ± 1.25			1.40 ± 0.58		
≥ 7 points	55	7.05 ± 3.47			0.52 ± 0.23		
*TNM staging*			7.113	< 0.001		18.225	< 0.001
cT1-2a	121	4.31 ± 2.01			4.02 ± 1.25		
cT2b and higher	77	8.52 ± 2.28			0.42 ± 0.28		

Data are shown as means ± SD. Result of student’s t-test. *P* < 0.05 is considered to be significant

### Serum miR-148a-3p increases while miR-485-5p decreases in postoperative recurrence compared with the non-recurrence group

**Fig. 1:**
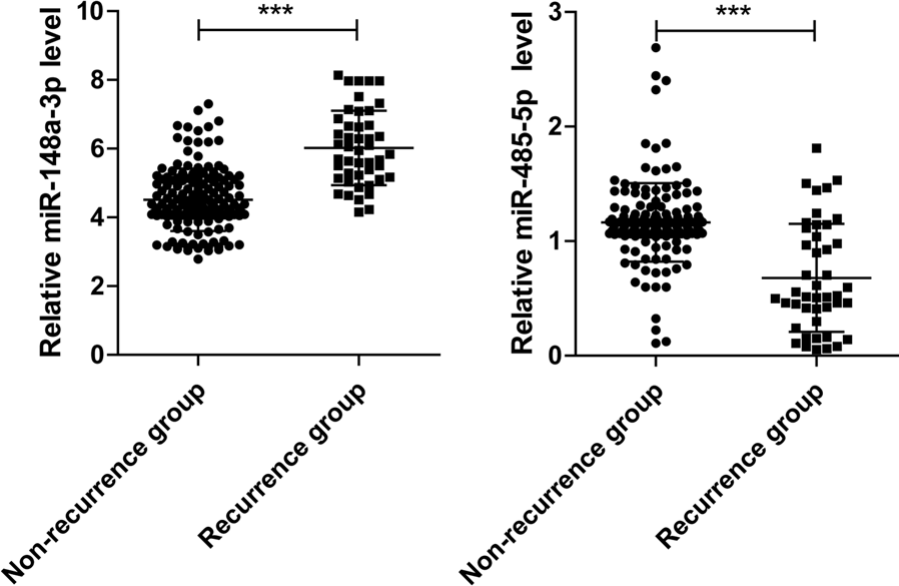
Dot plots showing increased miR-148a-3p and decreased miR-485-5p in the recurrence group compared with the non-recurrence group. The levels of serum miR-148a-3p and miR-485-5p expression in non-recurrence group (n = 153) and recurrence group (n = 45) was determined via qRT-PCR analysis

We next examined the possible correlation between serum levels of miR-148a-3p and miR-485-5p and the recurrence of prostate cancer. Serum levels of miR-148a-3p in the recurrence group (5.97 ± 0.18) were significantly higher than that of the non-recurrence group (4.53 ± 0.07) (t = 8.502, *P* < 0.001). In comparison, serum miR-485-5p levels in the recurrence group (0.27 ± 0.03) were lower than that of the non-recurrence group (1.22 ± 0.02) (t = 27.72, *P* < 0.001) (Fig. 1).

### Both miR-148a-3p and miR-485-5p demonstrate great potential as sensitive and specific biomarkers for prostate cancer

**Fig. 2:**
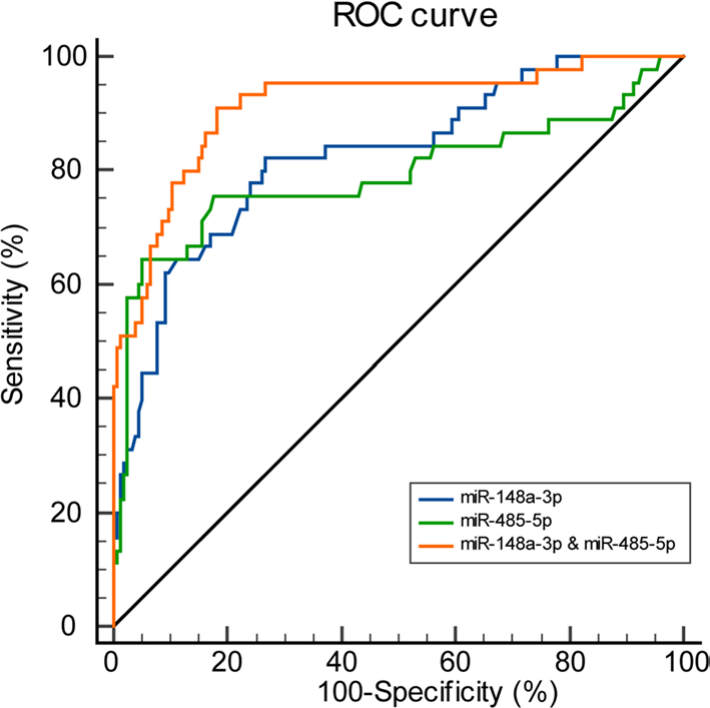
Receiver operating characteristic (ROC) curve analysis of the variations of miR-148a-3p and miR-485-5p in PCa patients. *P* < 0.05 was considered statistically significant

We then evaluated the sensitivity and specificity of using serum levels of miR-148a-3p and miR-485-5p to distinguish non-recurrence versus recurrence. We performed a receiver operating characteristic (ROC) curve analysis of the variations of serum levels of miR-148a-3p and miR-485-5p and calculated the area under the ROC curve (AUC) and optimal cut-off values (Fig. 2; Table 3). Both miR-148a-3p and miR-485-5p demonstrate significant sensitivity and specificity in distinguishing non-recurrence versus recurrence. Respectively, the AUC of recurrence in combined detection was greater than that of individuals detection (Z = 2.42, *P* = 0.0155; Z = 3.234, *P* = 0.0012). Thus, using the two molecules combined to predict prostate cancer recurrence was even more specific and sensitive than single detection.

**Table 3:** ROC curve analysis of the variations of miR-148a-3p and miR-485-5p in recurrent PCa

	AUC	95% CI	sensitivity	specificity	BCV	P
miR-148a-3p	0.825	0.765–0.875	82.22	73.2	5.01	< 0.0001
miR-485-5p	0.790	0.726–0.844	64.44	94.77	0.70	< 0.0001
miR-148a-3p& miR-485-5p	0.913	0.865–0.948	91.11	81.7	0.18	< 0.0001

AUC: area under the ROC curve; BCV: Best Critical Value; *P* < 0.05 was considered statistically significant

## Discussion

In the presented study, we examined the correlation between pre-surgery serum levels of miR-148a-3p and miR-485-5p and prostate cancer progression (indicated by Gleason score and TNM stages) and postoperative recurrence in 198 patients with prostate cancer who underwent surgery. We also evaluated the sensitivity and specificity of miR-148a-3p and miR-485-5p as diagnostic markers. Our results suggested that serum levels of miR-148a-3p positively correlated while miR-485-5p negatively correlated with Gleason score and TNM staging in PCa patients. Patients diagnosed with postoperative recurrence tend to have higher serum levels of miR-148a-3p and lower miR-485-5p. Besides, our ROC analysis suggested that miR-148a-3p and miR-485-5p combined detection are potentially sensitive and specific diagnostic biomarkers for PCa postoperative recurrence.

Despite the advances in prostate cancer treatments [26], prostate cancer (PCa) has always been a significant threat to male health. one of the reasons is the lack of early diagnosis methods and ways to precisely evaluate the severity of progression stages of prostate cancer, which is critical for deciding the appropriate treatment. Primarily, the goal of PCa diagnosis is to discover the earliest possible clinically significant PCa to maximize oncological outcomes and minimize functional side effects [26, 27]. Thus, efficient and sensitive screening using biomarkers of PCa is an ideal strategy. Although the addition of biomarkers such as serum prostate-specific antigen levels to prostate cancer diagnosis has increased diagnosis at relatively early stages (Gleason < 7), the current diagnostic algorithm still presents several limitations. Besides, the low sensitivity and specificity of SPA antigens still highlight the urgent demand for sensitive and specific biomarkers. microRNAs (miRNAs) could be perspective biomarkers of PCa. miRNAs are endogenous non-coding RNA regulating post-transcriptional gene expression, which participates in cell proliferation, differentiation, and apoptosis process [28]. The involvements of miRNAs in PCa have been widely reported both as diagnostic biomarkers [15, 29–31] and as therapeutic targets [6, 32]. In the presented study, we examined the potential of using two miRNAs, miR-148a-3p and miR-485-5p, to diagnose and evaluate prostate cancer. miR-148a-3p regulates myoblast differentiation into myotubes and accelerates the proliferation of myoblasts in the G1 phase of the cell cycle [33, 34]. It has also been shown to be related to cellular apoptosis and promotes tumor progression by protecting tumors from immune attack [35]. Our study showed that serum miR-148a-3p levels increased in late-stage prostate cancer, suggesting its positive correlation with prostate cancer progression, which agrees with the published tumor-promoting function of miR-148a-3p [19]. miR-485-5p, on the other hand, is a tumor suppressor gene in various malignant tumors [24]. Published studies showed that miR-485-5p induced multidrug resistance in cisplatin-resistant cell lines [25] and activated the protein kinase B signaling pathway in breast cancer cells [26] to suppress tumor progression. Our results suggested that serum miR-485-5p levels negatively correlated with prostate cancer progression with decreased values in late-stage prostate cancer patients, which recapitulate the published tumor repression function. Together, our study suggests the potential of miR-148a-3p and miR-485-5p to diagnose and evaluate prostate cancer.

Another significant difficulty in curing prostate cancer is the postoperative recurrence, which is the recurrence of prostate cancer cells after initial treatment has removed them. Prostate cancer is mainly treated with surgery combined with radiotherapy, chemotherapy, and endocrine therapy [36, 37]. Although it is considered a comprehensive treatment, recurrence and metastasis rate is still as high as more than 15%. Besides, due to the difficulties in diagnosing prostate cancer, recurrence is always hard to predict. In our study, we examined the potential to use serum levels of biomarkers measured pre-surgery to predict the probability of recurrence after treatment, allowing doctors to anticipate patients’ recovery better and be more prepared for potential recurrence. Our results suggested that patients who eventually got prostate cancer recurrence always had higher serum levels of miR-148a-3p and lower serum levels of miR-485-5p even before the initial treatment was performed, highlighting the possibility of using pre-surgery measures to anticipate recurrence. Besides, our ROC analysis indicates the high sensitivity and specificity of using pre-surgery miR-148a-3p and miR-485-5p levels to distinguish recurrence versus non-recurrence patients, suggesting the potential for predicting recurrence based on these two molecules. In addition, our results suggest that combining both pre-surgery serum levels of miR-148a-3p and miR-485-5p gives a significantly better prediction. Our studies suggest that measuring pre-surgery serum levels of miR-148a-3p and miR-485-5p may help with diagnosis, precisely evaluating cancer stages, and anticipating postoperative recurrence for PCa patients.

## Limitations

Although our study demonstrated promising biomarkers, there are still some limitations in this study. Limitations include (a) This study only enrolls patients with PCa from the Shaanxi area; thus, it may not fully represent PCa patients in the global trend. (b) Our study only focuses on two miRNAs, including miR-148a-3p and miR-485-5p. A comprehensive analysis using a high throughput technique to screen for the miRNAs profiles is still needed to identify diagnostic markers. (c) Our study only measures miR-148a-3p and miR-485-5p in a small population, and future study is needed to apply our findings to a large population to evaluate miR-148a-3p and miR-485-5p as biomarkers.

## Conclusion

In conclusion, our study demonstrated that pre-surgery serum levels of miR-148a-3p and miR-485-5p could be used as sensitive and specific biomarkers for diagnosis, evaluating cancer progression stages, and anticipating postoperative recurrence.

## Data Availability

The datasets used during the current study available from the corresponding author on request.

## Abbreviations

PCa: Prostate cancer
miRNAs: microRNAs
ROC: Receiver operating characteristic curve
